# Supplementary figures and images for: Nonlytic cellular release of hepatitis A virus requires dual capsid recruitment of the ESCRT-associated Bro1 domain proteins HD-PTP and ALIX
Source: PLoS Pathog. 2022 Aug 15;18(8):e1010543. doi: 10.1371/journal.ppat.1010543 (PMC9410543; doi:10.1371/journal.ppat.1010543)

**A**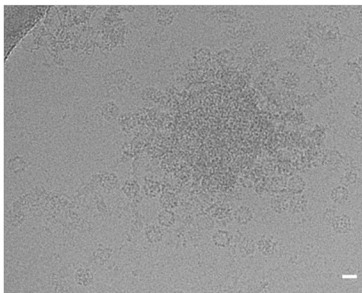**B**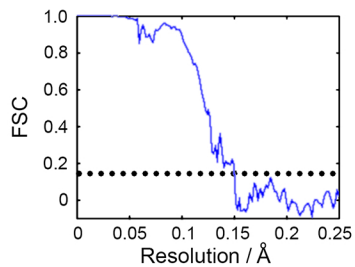**C**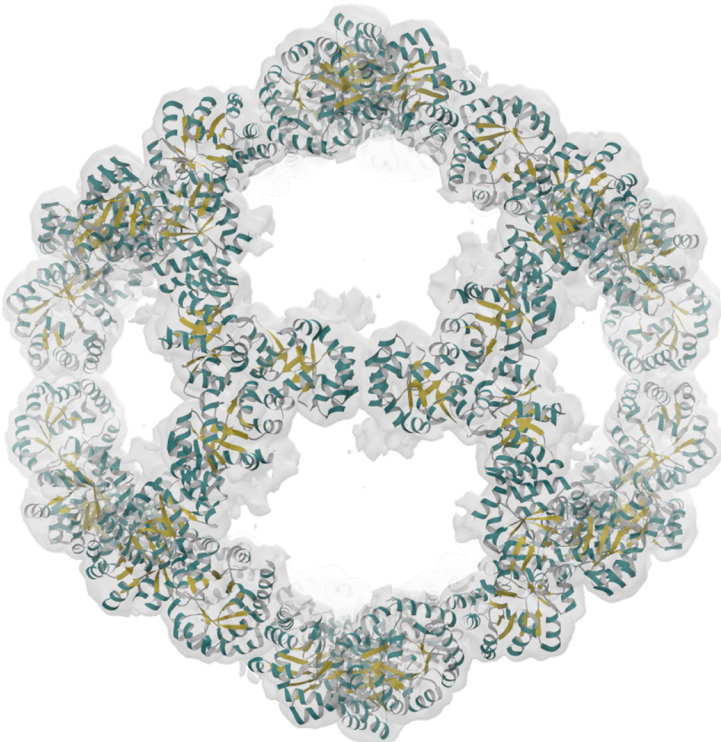**D**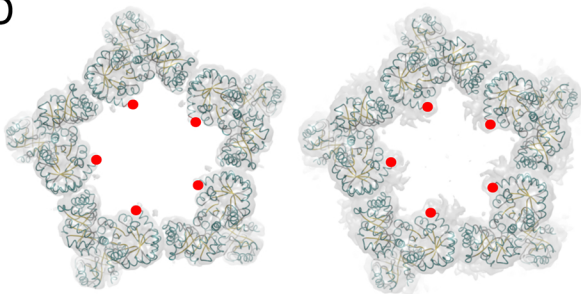**E**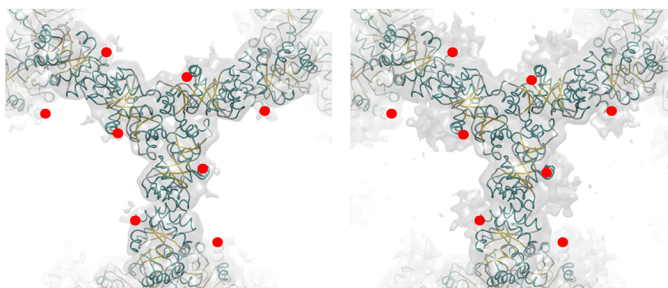**F**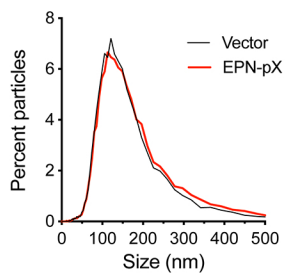**G**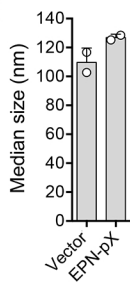**H**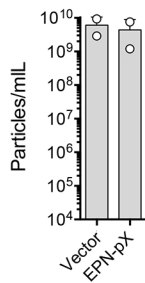

Supplement: S1 Fig — (A) Representative electron micrograph of extracellular fluids showing a burst vesicle. Scale bar = 200 Å. (B) Fourier shell correlation (FSC) plot showing attained resolution of 6.7 Å. (C) Overview of three-dimensional map with a rigid-body fitted to a previously published model of the EPN nanocage (PDB: 5KP9) in cartoon representation. Secondary structural elements are colored with helices in cyan and β-sheets in yellow. (D) Exterior views of the five-fold cage face at (left) high and (right) low contour levels showing additional density that may be consistent with pX. The C-terminus of the EPN sequence is indicated by the red dot. (E) Interior views of the three-fold nanocage axis, displayed as in panel D. (F) Laser-scattering video microscopy (NTA) estimates of the size distribution of extracellular particles in supernatant fluids of 293T cells 24 hr after transfection with EPN-pX (n = 22,114 particles) or empty vector (n = 18,038). (G) Median particle size measured by NTA in 2 independent transfection experiments. (H) NTA estimates of EV concentration in extracellular fluids. (PDF) [file ppat.1010543.s001.pdf]

**A**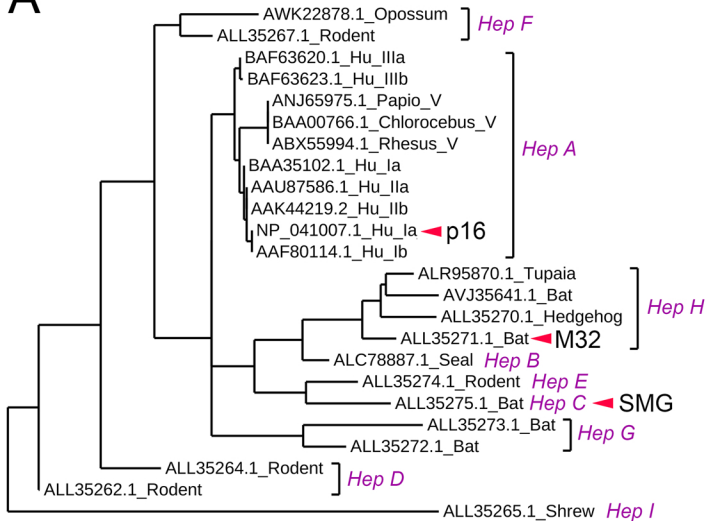**B**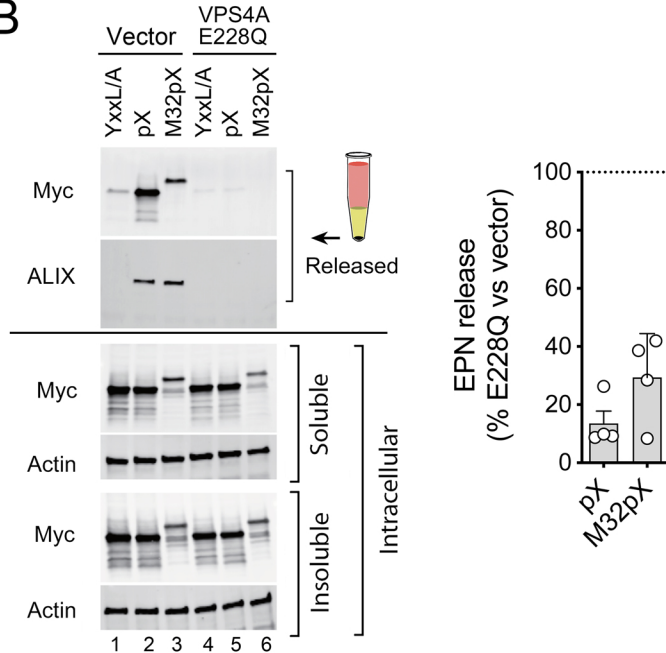

Supplement: S4 Fig — (A) Phylogenetic tree showing relatedness of amino acid sequences of pX in 9 recognized hepatovirus species infecting 18 different mammalian species (magenta font, “Hep” = Hepatovirus species). GenBank accession numbers and genotype of Hepatovirus A viruses are indicated. Arrows denote pX sequences studied as EPN fusions (Fig 3). (B) Immunoblots showing released particulate EPN-pX and EPN-M32pX is associated with ALIX. EPN-YxxL/A was included as a negative control. Cells were co-transfected with an empty vector (lanes 1–3) or vector expressing the dominant-negative VPS4A E228Q mutant (lanes 4–6) to demonstrate that release is ESCRT dependent. To the right is shown the mean normalized percent release of EPN-pX and EPN-M32pX in cells expressing VPSA E228Q versus empty vector, ± S.E.M., n = 4. (PDF) [file ppat.1010543.s004.pdf]

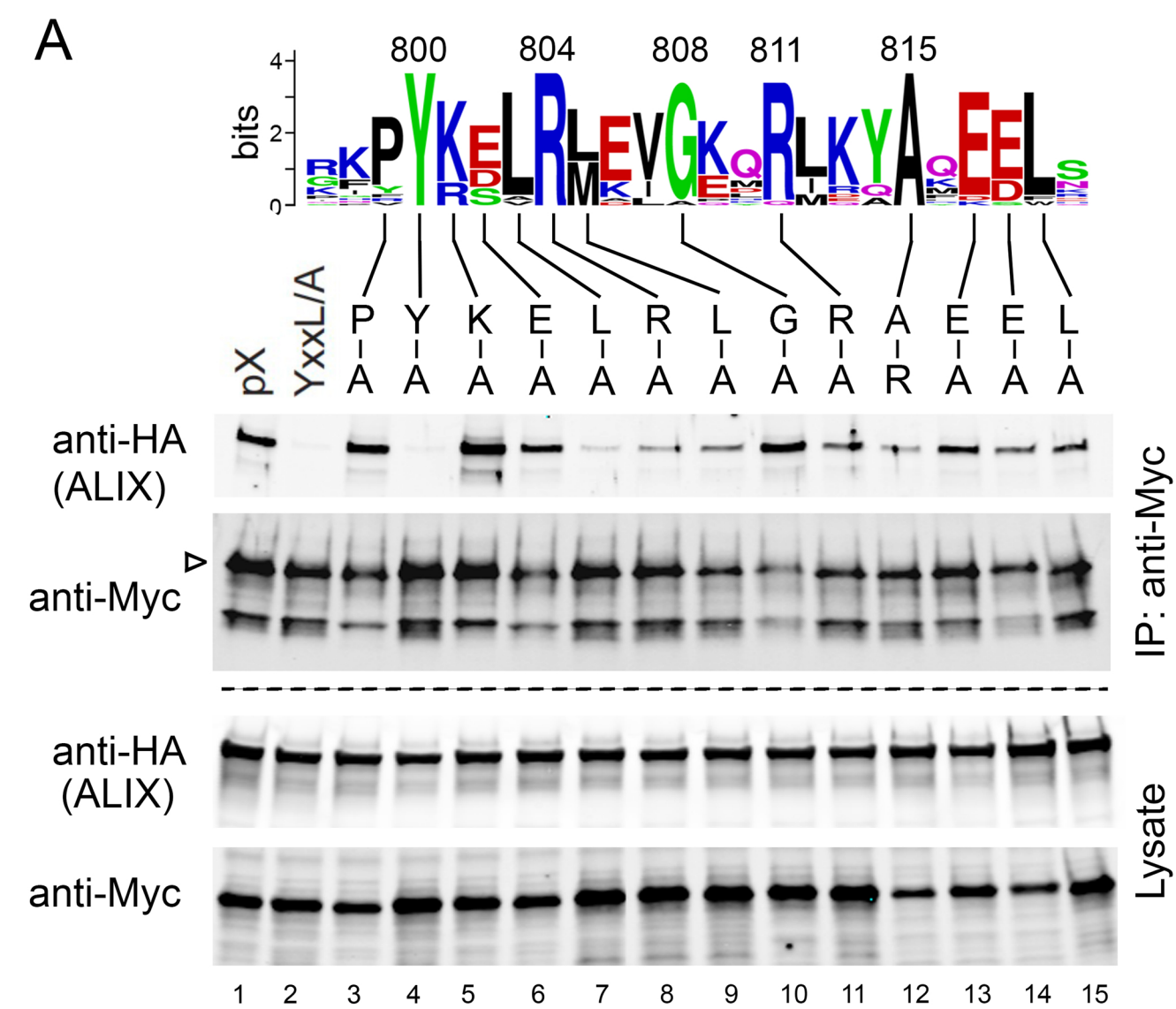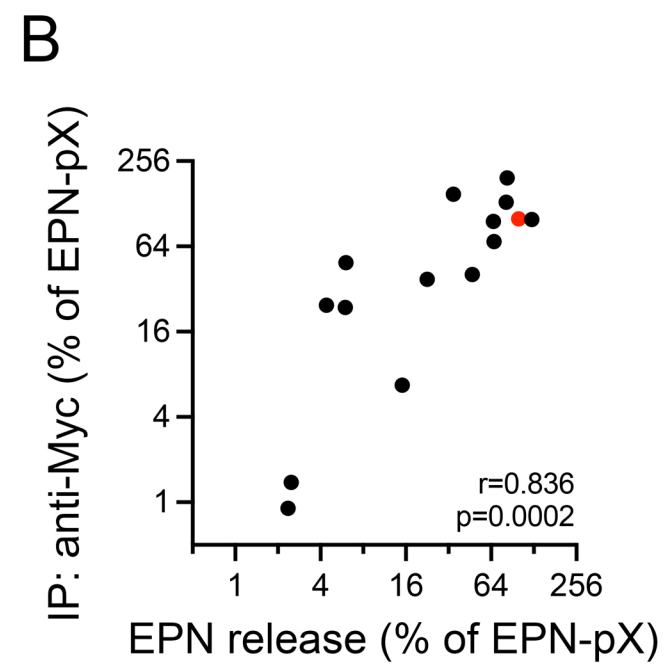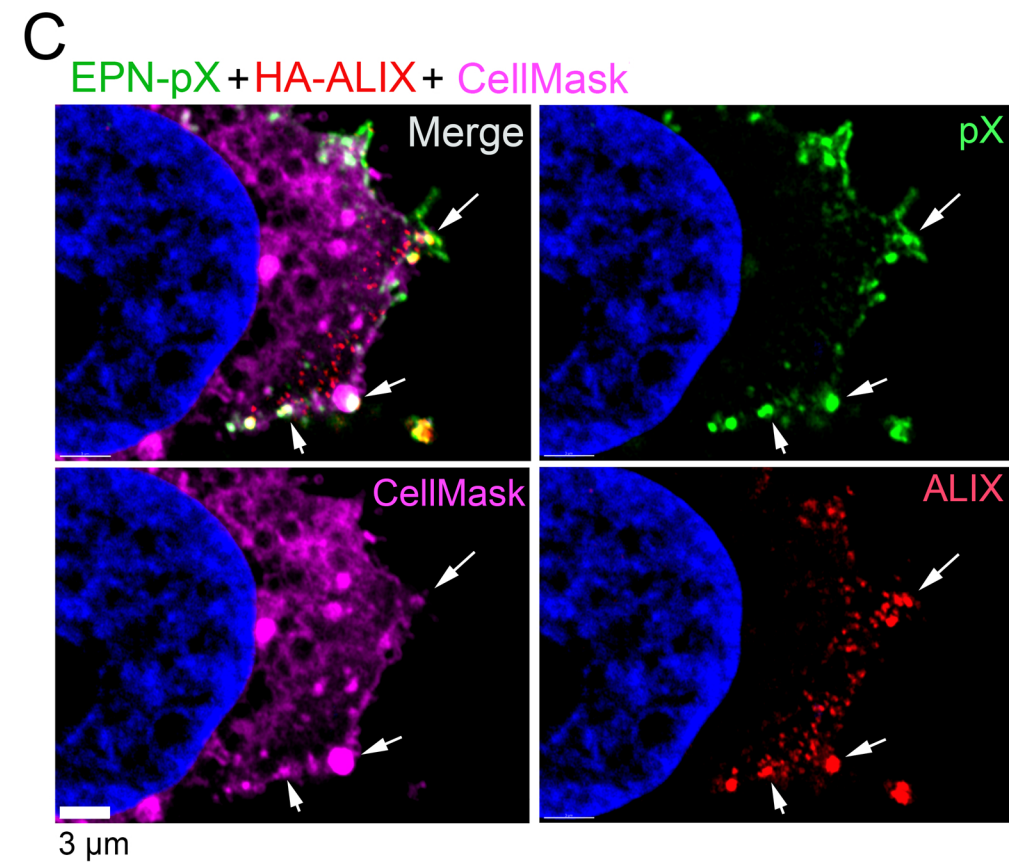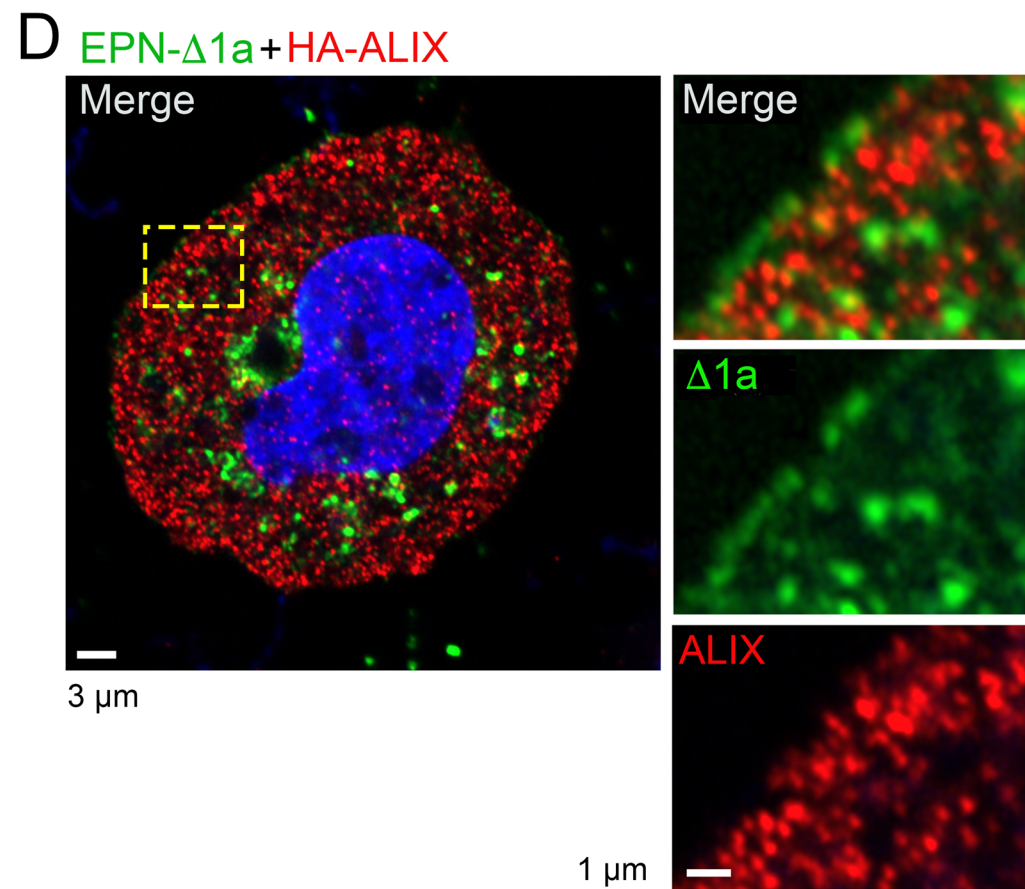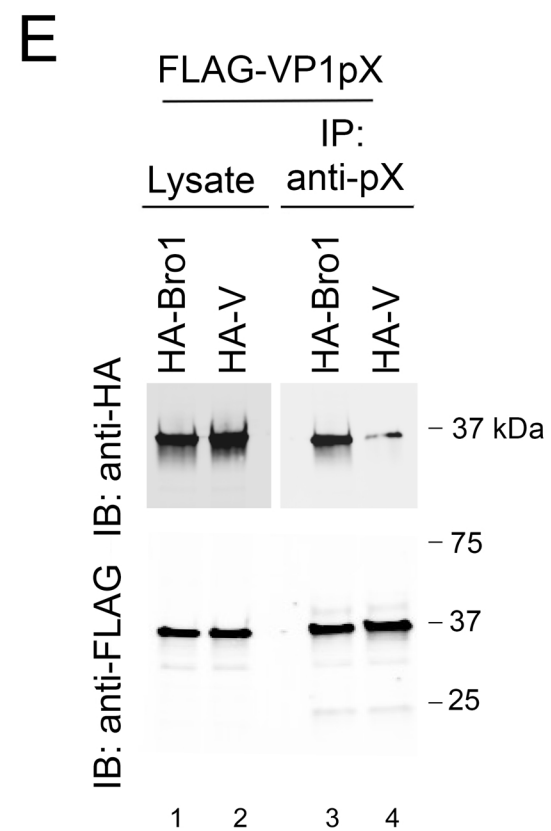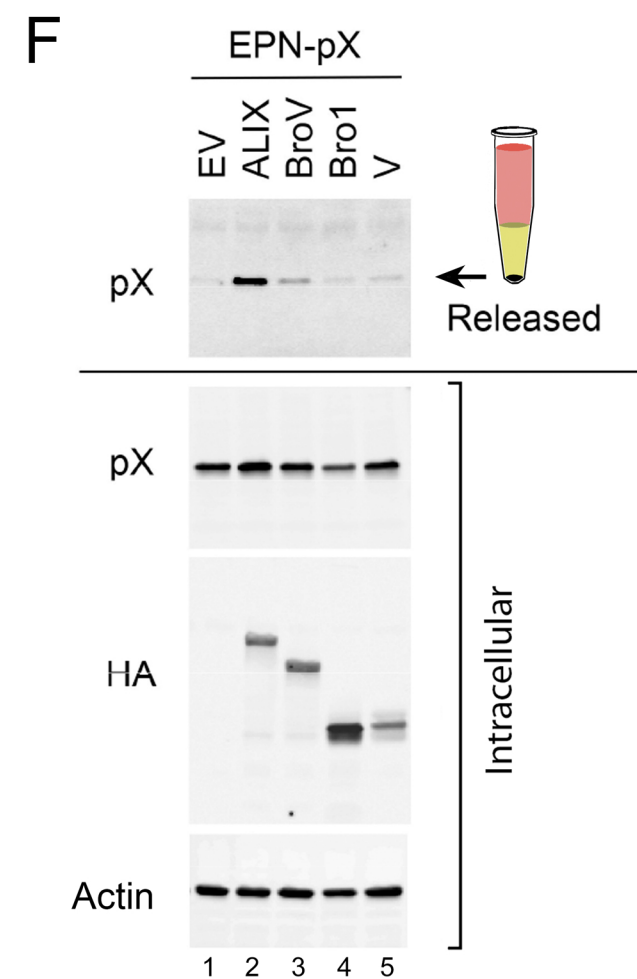

Supplement: S6 Fig — (A) Co-immunoprecipitation of HA-ALIX and EPN-pX or EPN-pX mutants containing single amino acid substitutions of conserved ExpD residues co-expressed in 293T cells. Lysates of 293T cells transfected with DNAs expressing the indicated nanocage proteins and HA-tagged ALIX were precipitated with anti-Myc antibody, then immunoblotted with anti-HA antibody. (B) Quantitative comparison of the efficiency of co-immunoprecipitation of EPN constructs with ALIX shown in panel A versus the efficiency of EPN release when fused to pX with mutations in the ExpD domain (Fig 3F). Data are means from 3 (EPN release) or 2 (co-immunoprecipitation) independent experiments, normalized to EPN-pX (100%, red symbol). (C) Merged and single-channel Zeiss Airyscan super-resolution fluorescent images of cells transfected with EPN-pX and HA-ALIX expression vectors, with labeling for pX (green) and HA (ALIX, red). Membranes were visualized by labeling with CellMask-647 (magenta). Nuclei were visualized by staining with Hoechst (blue). Extensive pX-ALIX colocalization is evident at buds on the plasma membrane (arrows). (D) Super-resolution images of a cell transfected with EPN-Δ1a and HA-ALIX expression vectors showing negligible Δ1a and ALIX colocalization. (E) Co-immunoprecipitation of HA-tagged Bro1 and V domains of ALIX (see Fig 4D) with FLAG-tagged VP1pX expressed in 293T cells. (F) Released extracellular and intracellular EPN-pX nanocage protein in 293T cells overexpressing ALIX or the indicated ALIX domain fragments. EV = empty vector. (PDF) [file ppat.1010543.s006.pdf]

**A** HAV + ALIX + HD-PTP

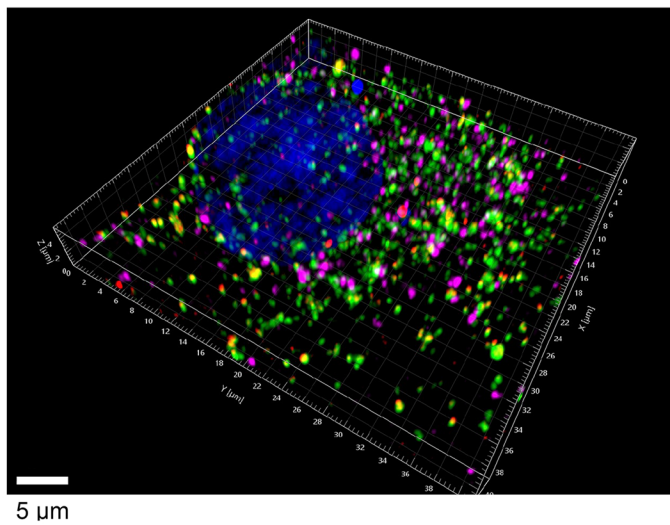

**B** HAV + ALIX + HD-PTP

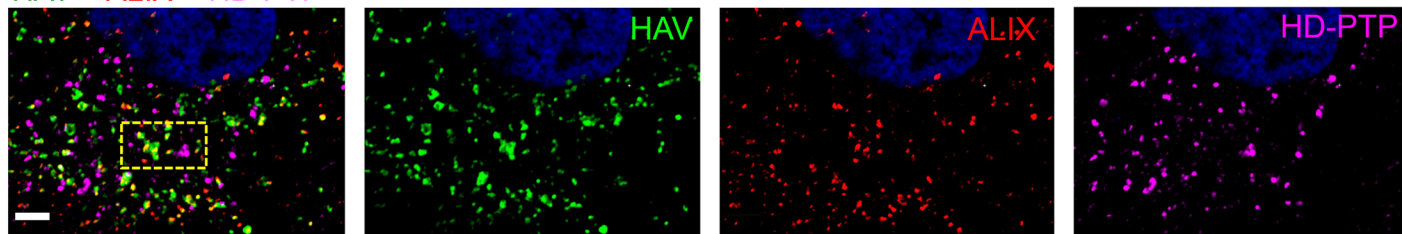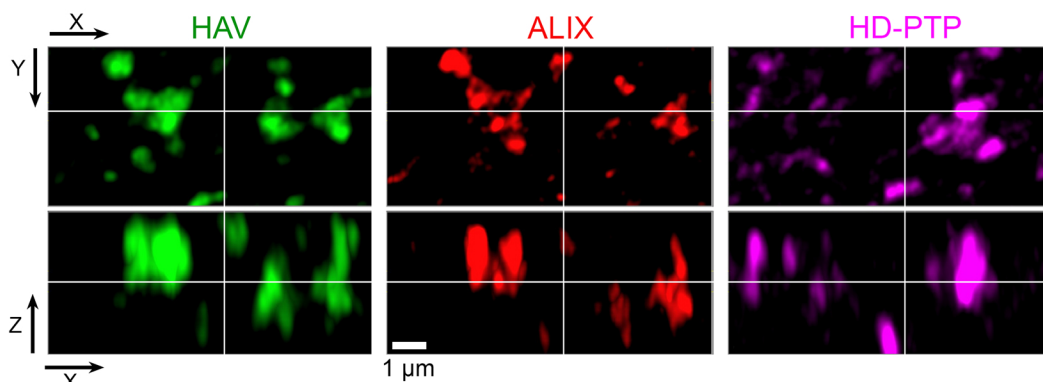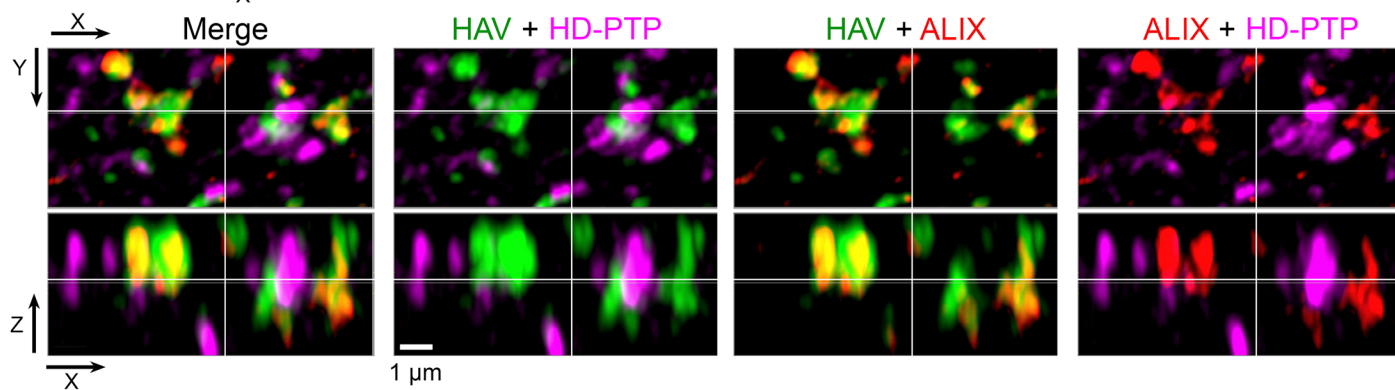

Supplement: S7 Fig — (A) Low magnification view and (B) super-resolution fluorescent microscopy images of a cell infected with 18f virus, demonstrating close proximity of viral antigens recognized by polyclonal human anti-HAV (‘HAV’, green), endogenous ALIX (red), and endogenous HD-PTP (magenta). High magnification single- and dual channel images of the region bounded by the dashed yellow box are shown below in three dimensions below. (PDF) [file ppat.1010543.s007.pdf]
