# Supplementary material for: Nonlytic cellular release of hepatitis A virus requires dual capsid recruitment of the ESCRT-associated Bro1 domain proteins HD-PTP and ALIX
Source: PLoS Pathog. 2022 Aug 15;18(8):e1010543. doi: 10.1371/journal.ppat.1010543 (PMC9410543; doi:10.1371/journal.ppat.1010543)
Supplement: S2 Fig — (A) (top) EPN-pX and EPN-p6Gag (previously named EPN01N [30]) constructs with single and dual deletions of the p6Gag late domains that bind TSG101 (PTAP) or ALIX (YPx3L) (p6GagΔ1 and p6GagΔL2, respectively). (bottom) Immunoblots showing extracellular release of EPN-pX compared with EPN-p6Gag with and without single or dual deletions of the p6Gag late domains. Deletion of either late domain ablates nanocage release, as shown previously [30]. (B) Absence of detectable extracellular release of EPN-VP2 which contains VP2 residues 130–195 fused to the EPN C-terminus, with and without Ala substitutions of the leading Tyr residue in both putative ALIX-interacting VP2 late domains (VP2-Y1,2A) [16]. EPN-pX was included as a positive control. (C) (top) EPN-pX nanocage constructs with and without the N-terminal HIV Gag myristoylation signal sequence. The ΔM2-6 and ΔM2-6-link constructs lack the p6Gag myristoylation signal (M2-6) that directs membrane association, while ΔM2-6-link also lacks a downstream linker sequence. (bottom) Myc immunoblots showing nanocage proteins released into extracellular fluids from transfected 293T cells (recovered after pelleting through a 20% sucrose cushion) and intracellular soluble and insoluble nanocage proteins expressed by the cells. Actin is shown as a loading control. (D) (top) EPN-pX constructs in which the Gag myristoylation signal (Gag residues 2–6) is replaced with peptide sequences from HAV VP4 (residues numbered according to the HAV ORF). (bottom) Myc immunoblots as in panel A. Lanes 6–10 show proteins released/expressed in cells treated with the N-myristoylation inhibitor IMP-1088. The inset shows a pX immunoblot of nanocage proteins released from cells transfected with EPN-pX (Gag myristoylation signal) and VP4-M7-11 (HAV VP4 sequence in lieu of the myristoylation signal), with and without IMP-1088 treatment, in an independent experiment. Note that synthesis of the HAV polyprotein can initiate at either the first or second AUG codo [file ppat.1010543.s002.pdf]

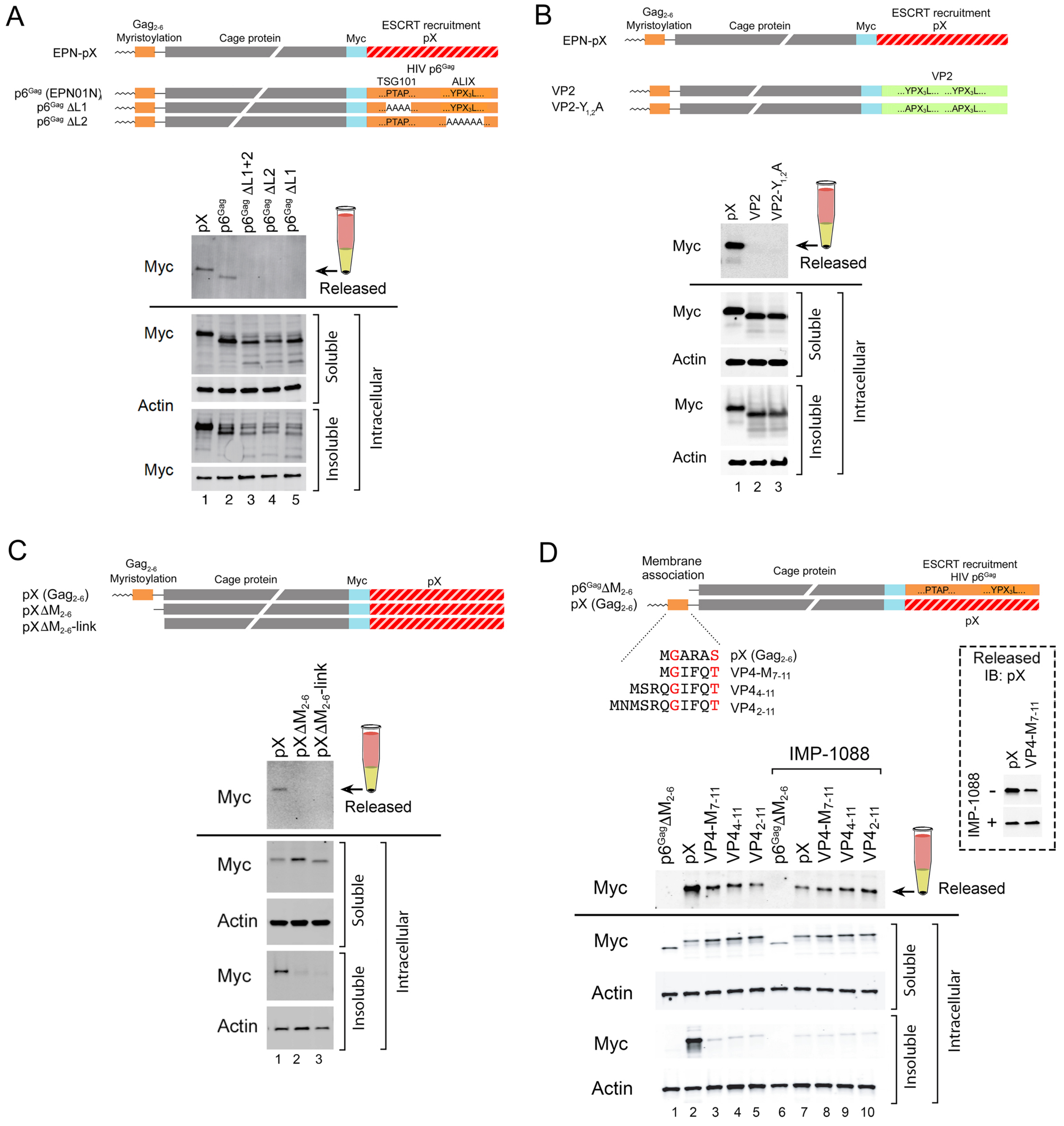
A
EPN-pX
Gag2-6 Myristoylation
Cage protein
Myc
ESCRT recruitment pX
HIV p6Gag
TSG101
ALIX
p6Gag (EPN01N)
p6Gag ΔL1
p6Gag ΔL2
...PTAP...
...YPX3L...
...AAAA...
...AAAAAA...
pX
p6Gag
p6Gag ΔL1+2
p6Gag ΔL2
p6Gag ΔL1
Myc
Released
Actin
Soluble
Insoluble
Intracellular
1 2 3 4 5
B
EPN-pX
VP2
VP2-Y1,2A
...APX3L...
Released
C
pX (Gag2-6)
pXΔM2-6
pXΔM2-6-link
D
Membrane association
p6GagΔM2-6
MGARAS pX (Gag2-6)
MGIFQT VP4-M7-11
MSRQGIFQT VP44-11
MNMSRQGIFQT VP42-11
Released IB: pX
IMP-1088
VP4-M7-11
VP44-11
VP42-11
1 2 3 4 5 6 7 8 9 10
