# Supplementary material for: Nonlytic cellular release of hepatitis A virus requires dual capsid recruitment of the ESCRT-associated Bro1 domain proteins HD-PTP and ALIX
Source: PLoS Pathog. 2022 Aug 15;18(8):e1010543. doi: 10.1371/journal.ppat.1010543 (PMC9410543; doi:10.1371/journal.ppat.1010543)
Supplement: S3 Fig — The alignment was constructed from 24 near genome-length hepatovirus sequences available in GenBank, recovered from 18 different mammalian species. Residues that are relatively well conserved are shown in red font, those less conserved in blue, followed by very poorly conserved residues in grey font. Residues that are absolutely conserved are underlined. Predicted VP1 and protein 2B sequences flanking pX are shaded in grey; the polyprotein cleavage sites have not been determined experimentally other than for Hepatovirus A. Genotype (gt) is noted for human and non-human primate (NHP)-derived strains. The alignment shows relatively high conservation of the export domain (ExpD, ++++++) which has the motif ‘Y[K/R]xLR[L/M]xxGxxRxxxA’, as well as a second conserved motif (‘[L/V]ESxVD’) within the pentamer assembly domain (PAD, ******). The ExpD sequence logo was generated from the 22 sequences above it with WebLogo version 2.8.2 (https://weblogo.berkeley.edu). Two bat Hepatovirus G viruses shown at the bottom have a large deletion in the pX region. *Bat virus pX sequences shown to mediate EPN secretion (see Fig 3B in the main manuscript). The alignment was produced by COBALT, with gap penalties of -11,-1 and a conservation setting of 3 bits (Papadopoulos, J.S., and Agarwala, R. Bioinformatics 23: 1073–1079, 2007), then manually adjusted. See S4A Fig for a phylogenetic tree. (PDF) [file ppat.1010543.s003.pdf]

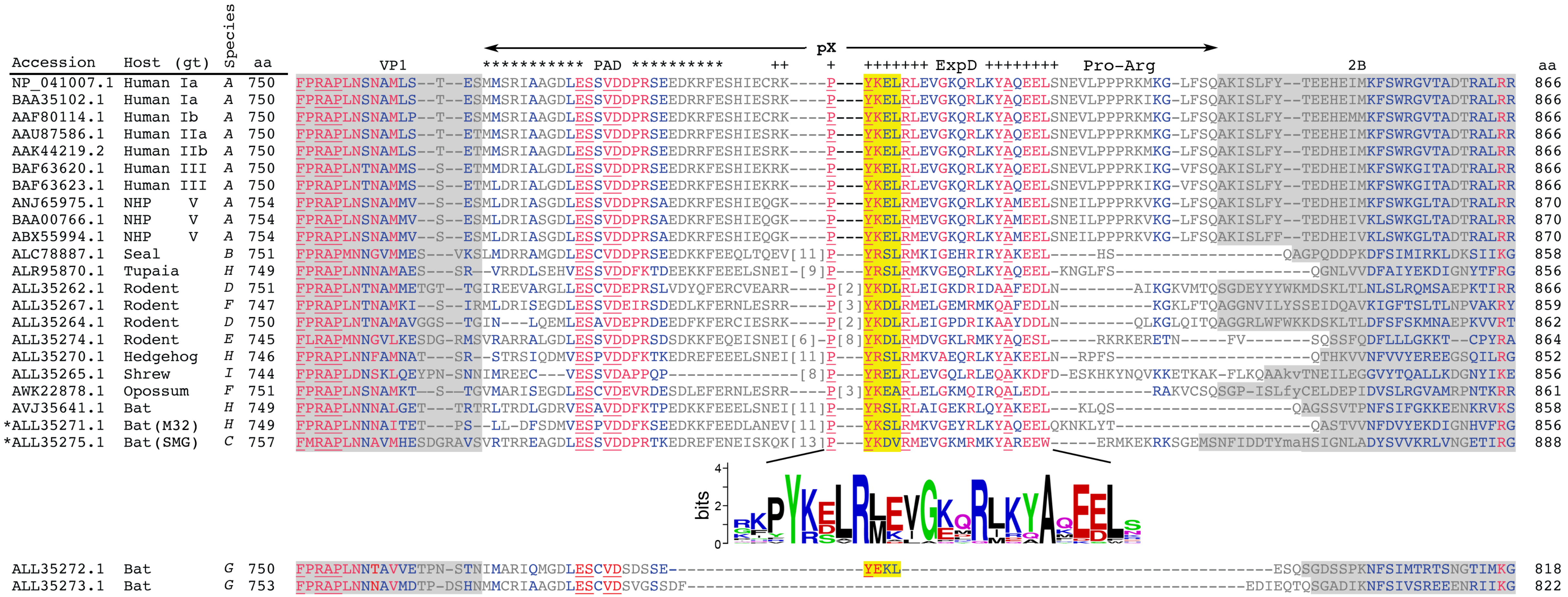

Accession Host (gt) Species aa
pX
VP1 *********** PAD ********** ++ + +++++++ ExpD ++++++++ Pro-Arg 2B aa
NP_041007.1 Human Ia A 750 FPRAPLNSNAMLS--T--ESMMSRIAAGDLESSVDDPRSEEDKRFESHIECRK----P---YKELRLEVGKQRLKYAQEELSNEVLPPPRKMKG-LFSQAKISLFY--TEEHEIMKFSWRGVTADTRALRR 866
BAA35102.1 Human Ia A 750 FPRAPLNSNAMLS--T--ESMMSRIAAGDLESSVDDPRSEEDRRFESHIESRK----P---YKELRLEVGKQRLKYAQEELSNEVLPPPRKIKG-LFSQAKISLFY--TEEHEIMKFSWRGVTADTRALRR 866
AAF80114.1 Human Ib A 750 FPRAPLNSNAMLP--T--ESMMSRIAAGDLESSVDDPRSEEDKRFESHIECRK----P---YKELRLEVGKQRLKYAQEELSNEVLPPPRKMKG-LFSQAKISLFY--TEEHEMMKFSWRGVTADTRALRR 866
AAU87586.1 Human IIa A 750 FPRAPLNSNAMLS--T--ETMMSRIAAGDLESSVDDPRSEEDRRFESHIESRK----P---YKELRLEVGKQRLKYAQEELSNEVLPPPRKMKG-LFSQAKISLFY--TEEHEIMKFSWRGVTADTRALRR 866
AAK44219.2 Human IIb A 750 FPRAPLNSNAMLS--T--ETMMSRIAAGDLESSVDDPRSEEDRRFESHIESRK----P---YKELRLEVGKQRLKYAQEELSNEVLPPPRKMKG-LFSQAKISLFY--TEDHEIMKFSWRGVTADTRALRR 866
BAF63620.1 Human III A 750 FPRAPLNTNAMMS--S--ETMMDRIALGDLESSVDDPRSEEDRKFESHIEKRK----P---YKELRLEVGKQRLKYAQEELSNEVLPPPRKIKG-VFSQAKISLFY--TEDHEIMKFSWKGITADTRALRR 866
BAF63623.1 Human III A 750 FPRAPLNTNAMMS--S--ETMLDRIALGDLESSVDDPRSEEDRKFESHIEKRK----P---YKELRLEVGKQRLKYAQEELSNEVLPPPRKIKG-VFSQAKISLFY--TEDHEIMKFSWKGITADTRALRR 866
ANJ65975.1 NHP V A 754 FPRAPLNSNAMMV--S--ESMLDRIASGDLESSVDDPRSAEDKRFESHIEQGK----P---YKELRMEVGKQRLKYAMEELSNEILPPPRKVKG-LFSQAKISLFY--TEDHEIMKFSWKGLTADTRALRR 870
BAA00766.1 NHP V A 754 FPRAPLNSNAMMV--S--ESMLDRIASGDLESSVDDPRSAEDKRFESHIEQGK----P---YKELRMEVGKQRLKYAMEELSNEILPPPRKVKG-LFSQAKISLFY--TEDHEIVKLSWKGLTADTRALRR 870
ABX55994.1 NHP V A 754 FPRAPLNSNAMMV--S--ESMLDRIASGDLESSVDDPRSAEDKRFESHIEQGK----P---YKELRMEVGKQRLKYAMEELSNEILPPPRKVKG-LFSQAKISLFF--TEDHEIVKLSWKGLTADTRALRR 870
ALC78887.1 Seal B 751 FPRAPMNNGVMMES---VKSLMDRRAMGDLESCVDDPRSEEDKKFEEQLTQEV[11]P---YRSLRMKIGEHRIRYAKEEL-----HS-----------------QAGPQDDPKDFSIMIRKLDKSIIKG 858
ALR95870.1 Tupaia H 749 FPRAPLNNNAMAES---SR--VRRDLSEHVESSVDDFKTDEEKKFEEELSNEI-[9]P---YRSLRMKVGEQRLKYAQEEL-KNGLFS--------------------QGNLVVDFAIYEKDIGNYTFRG 856
ALL35262.1 Rodent D 751 FPRAPLNTNAMMETGT--TGIREEVARGLLESCVDEPRSLVDYQFERCVEARR----P[2]YKDLRLEIGKDRIDAAFEDLN--------AIKGKVMTQSGDEYYYWKMDSKLTLNLSLRQMSAEPKTIRR 866
ALL35267.1 Rodent F 747 FPRAPLNTNAMKI--S--IRMLDRISEGDLESSVDEIRSDEDLKFERNLESRR----P[3]YKDLRMELGEMRMKQAFEDLN----------KGKLFTQAGGNVILYSSEIDQAVKIGFTSLTLNPVAKRY 859
ALL35264.1 Rodent D 750 FPRAPLNTNAMAVGGS--TGIN---LQEMLESAVDEPRDEEDFKFERCIESRK----P[2]YKDLRLEIGPDRIKAAYDDLN--------QLKGLQITQAGGRLWFWKKDSKLTLDFSFSKMNAEPKVVRT 862
ALL35274.1 Rodent E 745 FLRAPMNNGVLKESDG-RMSVRARRALGDLESSVDDPRSDSDKKFEQEISNEI[6]-P[8]YKDLRMDVGKLRMKYAQESL-----RKRKERETN-----FV-------SQSSFQDFLLLGKKT--CPYRA 864
ALL35270.1 Hedgehog H 746 FPRAPLNFAMNAT---SR--STRSIQDMVESPVDDFKTKEDREFEEELSNEI[11]P---YRSLRMKVAEQRLKYAKEELN--RPFS--------------------QTHKVVNFVVYEREEGSQILRG 852
ALL35265.1 Shrew I 744 FPRAPLDNSKLQEYPN-SNNIMREEC---VESSVDAPPQP-------------[8]P---YRELRLEVGQLRLEQAKKDFD-ESKHKYNQVKKETKAK-FLKQAAkvTNEILEGGVYTQALLKDGNYIKE 856
AWK22878.1 Opossum F 751 FPRAPLNSNAMKT--S--TGVMARISEGDLESCVDEVRDESDLEFERNLESRR----P[3]YKEARLELGKMQIRQALEDL------------RAKVCSQSGP-ISLfyCELDEPIDVSLRGVAMRPNTKRR 861
AVJ35641.1 Bat H 749 FPRAPLNNNALGET---TRTRLTRDLGDRVESAVDDFKTPEDKKFEEELSNEI[11]P---YRSLRLAIGEKRLQYAKEEL---KLQS-------------------QAGSSVTPNFSIFGKKEENKRVKS 858
*ALL35271.1 Bat(M32) H 749 FPRAPLNNNAITET---PS--LL-DFSDMVESPVDDFKSEEDKKFEEDLANEV[11]P---YKSLRMKVGEYRLKYAQEELQKNKLYT--------------------QASTVVNFDVYEKDIGNHVFRG 856
*ALL35275.1 Bat(SMG) C 757 FMRAPLNNAVMHESDGRAVSVRTRREAGDLESSVDDPRTKEDREFENEISKQK[13]P---YKDVRMEVGKMRMKYAREEW-----ERMKEKRKSGEMSNFIDDTYmaHSIGNLADYSVVKRLVNGETIRG 888
bits
4
2
0
ALL35272.1 Bat G 750 FPRAPLNNTAVVETPN-STNIMARIQMGDLESCVDSDSSE--------------------YEKL---------------------------------ESQSGDSSPKNFSIMTRTSNGTIMKG 818
ALL35273.1 Bat G 753 FPRAPLNNNAVMDTP-DSHNMMCRIAAGDLESCVDSVGSSDF--EDIEQTQSGADIKNFSIVSREENRIIKG 822
