# Supplementary material for: Nonlytic cellular release of hepatitis A virus requires dual capsid recruitment of the ESCRT-associated Bro1 domain proteins HD-PTP and ALIX
Source: PLoS Pathog. 2022 Aug 15;18(8):e1010543. doi: 10.1371/journal.ppat.1010543 (PMC9410543; doi:10.1371/journal.ppat.1010543)
Supplement: S5 Fig — (A) pX sequences in expression vectors used for LFQ analysis of pX-interacting proteins. EPN-PAD contains only the pentamer assembly domain (PAD) of pX. (B) Anti-Myc immunoblot showing triplicate protein precipitates subjected to proteomics analysis from 293T cells expressing the EPN-pX and EPN-PAD constructs. (C) Mean LFQ intensities of pX peptides in both protein precipitates (n = 3 samples of each precipitate, each with 2 technical replicates). Intensities of peptides identified in the EPN-PAD sample (common to both EPN-pX and EPN-PAD) are shown in solid black bars; intensities of peptides unique to EPN-pX are shown in red. (D) Percentage of the pX sequence covered by peptides found in the EPN-PAD and EPN-pX samples. (E) Spectral counts of selected proteins identified in the proteomics samples. C-term pX = pX sequence uniquely present in EPN-pX and absent in EPN-PAD. (F) Peptide coverage of selected proteins identified in the proteomics samples. (G) Cellular component of all 349 proteins enriched over 2-fold in EPN-pX compared to EPN-PAD precipitates (FDR<0.05). Significance for protein clustering with each component is plotted on the right axis (hatched bars). (PDF) [file ppat.1010543.s005.pdf]

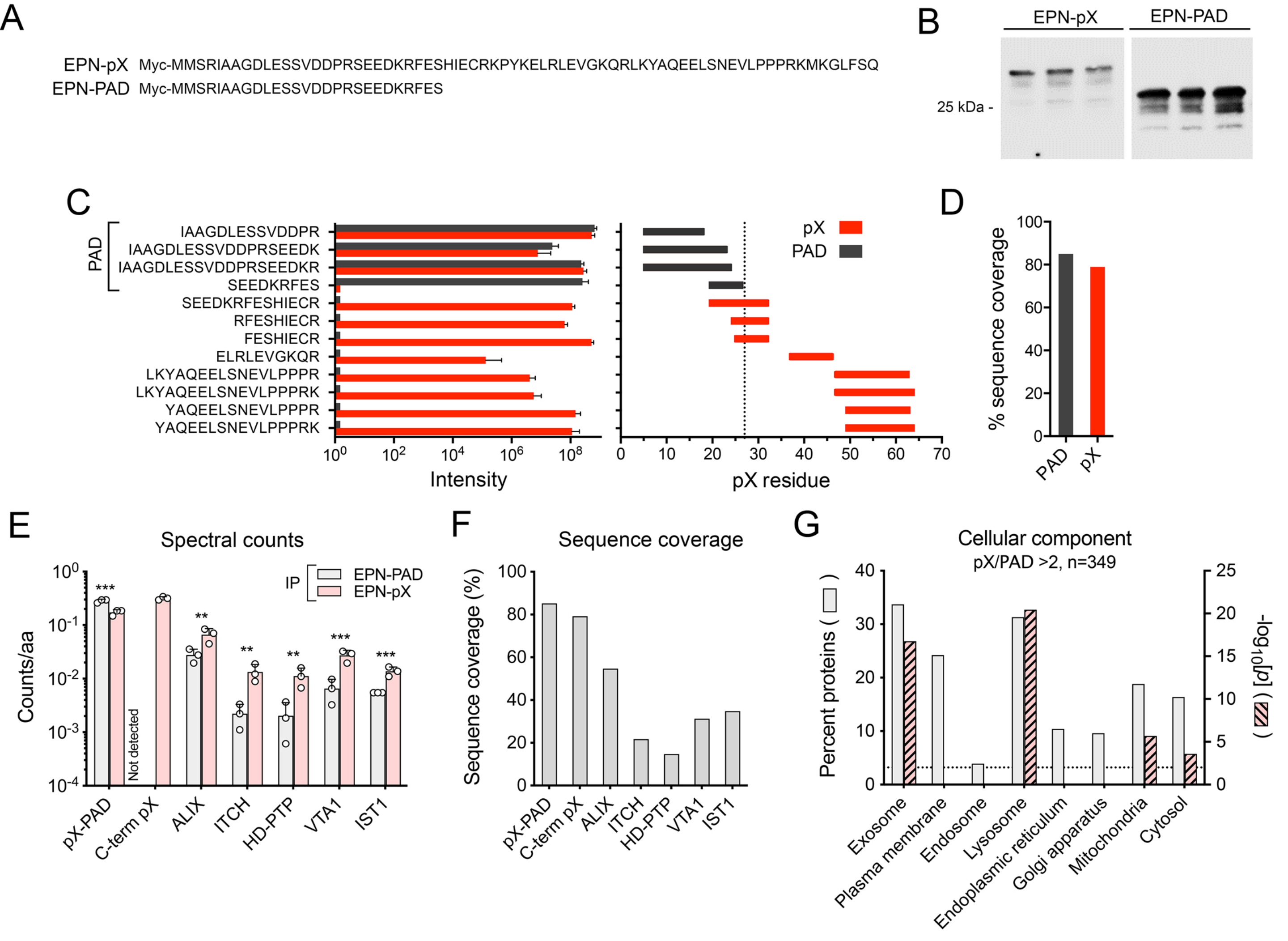

A
EPN-pX Myc-MMSRIAAGDLESSVDDPRSEEDKRFESHIECRKPYKELRLEVGKQRLKYAQEELSNEVLPPPRKMKGLFSQ
EPN-PAD Myc-MMSRIAAGDLESSVDDPRSEEDKRFES
B
EPN-pX
EPN-PAD
25 kDa -
C
PAD
IAAGDLESSVDDPR
IAAGDLESSVDDPRSEEDK
IAAGDLESSVDDPRSEEDKR
SEEDKRFES
SEEDKRFESHIECR
RFESHIECR
FESHIECR
ELRLEVGKQR
LKYAQEELSNEVLPPPR
LKYAQEELSNEVLPPPRK
YAQEELSNEVLPPPR
YAQEELSNEVLPPPRK
Intensity
pX residue
pX
PAD
D
% sequence coverage
PAD
pX
E
Spectral counts
Counts/aa
IP
EPN-PAD
EPN-pX
Not detected
pX-PAD
C-term pX
ALIX
ITCH
HD-PTP
VTA1
IST1
F
Sequence coverage
Sequence coverage (%)
pX-PAD
C-term pX
ALIX
ITCH
HD-PTP
VTA1
IST1
G
Cellular component
pX/PAD >2, n=349
Percent proteins
-log10[p]
Exosome
Plasma membrane
Endosome
Lysosome
Endoplasmic reticulum
Golgi apparatus
Mitochondria
Cytosol
