# Supplementary material for: Nonlytic cellular release of hepatitis A virus requires dual capsid recruitment of the ESCRT-associated Bro1 domain proteins HD-PTP and ALIX
Source: PLoS Pathog. 2022 Aug 15;18(8):e1010543. doi: 10.1371/journal.ppat.1010543 (PMC9410543; doi:10.1371/journal.ppat.1010543)
Supplement: S2 Table — (PDF) [file ppat.1010543.s009.pdf]

Table S2. Oligonucleotides used in this study.

Primer	5'-Sequence-3'	Purpose
pX-F	ATTCGTGGCTGCACCGAACAGAAGCTGATCTCCGAAGAAGACCTGATGATGAGCAGAATTG	Replacing HIV p6^{Gag} sequence with full-length, N-terminus (Pentamer domain), C-terminus (ΔPAD), secretion region (SecD) of HAV pX, or YPX_3L tandem late domains containing VP2 fragment
pXΔPAD-F	ATTCGTGGCTGCACCGAACAGAAGCTGATCTCCGAAGAAGACCTGATGCATATAGAATGCAGGAAG	
pXΔ123-R	GGCAGAGGGAAAAAGATCTGCTAGCAAGCTTCTCGAGTCAACTCTCAAATCTTTTATCT	
pX/Δ1/Δ2/Δ3-R2	GGCAGAGGGAAAAAGATCTGCTAGCAAGCTTCTCGAGTCATTGTGAAAACAGTCC	
SecD-F	ATTCGTGGCTGCACCGAACAGAAGCTGATCTCCGAAGAAGACCTGAGGAAGCCATAT	
SecD-R	GGCAGAGGGAAAAAGATCTGCTAGCAAGCTTCTCGAGTCATGACAATTCTTCC	
VP2LD-F	ATTCGTGGCTGCACCGAACAGAAGCTGATCTCCGAAGAAGACCTGCCTGGTGACCAGAGCTAT	
VP2LD-R	GGCAGAGGGAAAAAGATCTGCTAGCAAGCTTCTCGAGTCATCCTGTCCCAATATTTAATTCTGACC	
EPN01-Seq	GAATGGTTCAAAGCGGGC	
ΔHIVM2-5 F	TATATGGTACCATGGGTTCTAAATCTGGCTCCGGC	Deleting Myristoylation signal from EPN-pX
ΔHIVM2-link F	TATATGGTACCATGAAGATCGAAGAGCTGTTCAAGA	
ΔHIVM2-5 R	TGGATGGTACCTCCGGATCGATAATTCTAG	
pXΔ1a-F	TGAGGGATCCGAAGTTGGGAAACAAAGACTCAAGTATGC	
pXΔ1a-R	TGAGGGATCCACTCTCAAATCTTTTATCTTCCTCTGATCTAGG	
pXΔ2a-F	TGAGGGATCCGAAGAATTGTCAAATGAAGTACTTCCACC	
pXΔ2a-R	TGAGGGATCCTAATCTCAGTTCTTTATATGGCTTCCTGC	
EPN01-seq2	AAATGGGCGGTAGGCGTGTA	
VP4/7-11 F	GAATTATCGATCCGGAGGTACCATGGGTATTTTCCAGACTGGTTCTAAATCTGGCTCCGGCTCTG	Cloning sequences with different length of HAV VP4 to EPN-pX
VP4/7-11 R	CAGAGCCGGAGCCAGATTTAGAACCAGTCTGGAAAATACCCATGGTACCTCCGGATCGATAATTC	
VP4/3-11 F	GAATTATCGATCCGGAGGTACCATGTCTAGACAAGGTATTTTCCAGACTGGTTCTA	
VP4/3-11 R	TAGAACCAGTCTGGAAAATACCTTGTCTAGACATGGTACCTCCGGATCGATAATTC	
VP4/1-11 F	GAATTATCGATCCGGAGGTACCATGAACATGTCTAGACAAGGTATTTTCCAGACTGGTTCTA	
VP4/1-11 R	TAGAACCAGTCTGGAAAATACCTTGTCTAGACATGTTCATGGTACCTCCGGATCGATAATTC	
YxxL/A-F	GAGAGTCATATAGAATGCAGGAAGCCAGCTGCAGCAGCGAGATTAGAAGTTGGGAAACAAAGACTC	Constructing Ala substitutions at the YxxL motif in EPN-pX
YxxL/A-R	GAGTCTTTGTTTCCCAACTTCTAATCTCGCTGCTGCAGCTGGCTTCCTGCATTCTATATGACTCTC	
KQ1/A-F	GCCATATAAAGAACTGAGATTAGAAGTTGCGGCAGCAAGACTCAAGTATGCTCAGGAAGAATTG	Constructing Ala substitutions at the KFERQ-like motifs in EPN-pX
KQ1/A-R	CAATTCTTCCTGAGCATACTTGAGTCTTGCTGCCGCAACTTCTAATCTCAGTTCTTTATATGGC	
KQ2/A-F	GATTAGAAGTTGGGAAACAAAGACTCAAGGCTGCTGCGGAAGAATTGTCAAATGAAGTACTTCCACC	

KQ2/A-R	GGTGGAAGTACTTCATTTGACAATTCTTCCGCAGCAGCCTTGAGTCTTTGTTTCCCAACTTCTAATC	
KQ1,2/A-F	GAAGTTGCGGCAGCAAGACTCAAGGCTGCTGCGGAAGAATTGTCAAATGAAGTACTTCCACC	
KQ1,2/A-R	GGTGGAAGTACTTCATTTGACAATTCTTCCGCAGCAGCCTTGAGTCTTGCTGCCGCAACTTC	
ALIX1-F	CCCACCATGTACCCATACGATGTTCCAGATTACGCTatggcgacattcatctcggt	Inserting coding sequence of ALIX into pCMV-HA
ALIX65-F	CCCACCATGTACCCATACGATGTTCCAGATTACGCTctcgagacgctcctgag	
ALIX135-F	CCCACCATGTACCCATACGATGTTCCAGATTACGCTgcagcagaacagaacctgg	
ALIX210-F	CCCACCATGTACCCATACGATGTTCCAGATTACGCTgatgccatcatagctaaattggc	
ALIX361-F	CCCACCATGTACCCATACGATGTTCCAGATTACGCTGTGTCAGTACAGCAGTCTTTGG	
ALIX360-R*	TCGACCGAATTCGGGCCTCCATGGCCATAAGTCAGGGAACCATCTTCTCAAACAGATC	
ALIX702-R*	TCGACCGAATTCGGGCCTCCATGGCCATAAGTCATCTTTCTGTCTTCCGTGCAA	
ALIX868-R*	TCGACCGAATTCGGGCCTCCATGGCCATAAGTCACTGCTGTGGATAGTAAGACTGC	
p16-SacI	CCCACCATGTACCCATACGATGTTCCAGATTACGCTGAGCTCCATTGAACTCAAA	Cloning HAV p16 strain fragment from SacI to EcoRI into pCMV-HA for YxxL/A construction
p16-EcoRI	TCGACCGAATTCGGGCCTCCATGGCCATAAGCTCCATGAATTCAGTCATGT	
p16/VP1-seqf	CACTGGATGGTTTGGGAGAC	
p16/3B-seqr	TTACACCATGATATACCCCTTCA	

*Red letters = stop codon
