# Supplementary material for: Nonlytic cellular release of hepatitis A virus requires dual capsid recruitment of the ESCRT-associated Bro1 domain proteins HD-PTP and ALIX
Source: PLoS Pathog. 2022 Aug 15;18(8):e1010543. doi: 10.1371/journal.ppat.1010543 (PMC9410543; doi:10.1371/journal.ppat.1010543)
Supplement: S3 Table — RRID: Research Resource Identification Portal, https://scicrunch.org/resources. (PDF) [file ppat.1010543.s010.pdf]

Table S3. Antibodies used in this study.

Antibody	Source	RRID
Mouse anti-Myc, clone 4A6	EMD Millipore Cat# 05-724	RRID:AB_568800
Rabbit anti-HA-Tag clone C29F4	Cell Signaling Cat# 3724	RRID:AB_1549585
Rat anti-HA clone 3F10	Roche Cat# 11867423001	RRID:AB_390918
Mouse anti- DYKDDDDK Tag	Cell Signaling Cat# 8146	RRID:AB_10950495
Mouse anti-ALIX-488 clone 1A12	Santa Cruz Cat# sc-53540	RRID:AB_673819
Rabbit anti-ALIX clone E6P9B	Cell Signaling Cat# 92880	RRID:AB_2800192
Rabbit anti-PTPN23 (HD-PTP)	Proteintech Cat# 10472-1-AP	RRID:AB_2173382
Mouse anti-LAMP1 clone D401S	Cell Signaling Cat# 15665	RRID:AB_2798750
Mouse anti-HAV clone K24F2	Commonwealth Serum Laboratories	RRID:AB_2868526
Guinea pig anti-VP1 #501	Gift from David Sangar	N/A
Human polyclonal anti-HAV JC	Lemon laboratory [1]	RRID:AB_2868527
Rabbit anti-GLuc	New England Biolabs Cat# E8023S	RRID:AB_1929564
Rabbit anti-Actin	Sigma-Aldrich Cat# A2066	RRID:AB_476693
Mouse anti-Actin clone C-2	Santa Cruz Cat# sc-8432	RRID:AB_626630
